# A Quality Improvement Project to Assess if Preoperative Trauma Patients Are Receiving Their Prescribed Medications Preoperatively

**DOI:** 10.7759/cureus.31928

**Published:** 2022-11-27

**Authors:** Ashwin Bhadresha, Sabir Hossain, Srinath Ranjit, Smita Singh, Shobhana Chhetri

**Affiliations:** 1 Trauma and Orthopedics, The Royal London Hospital, London, GBR; 2 Internal Medicine, Basildon University Hospital, London, GBR; 3 Trauma and Orthopedics, Whittington Hospital, London, GBR; 4 Anesthetics, Basildon University Hospital, London, GBR

**Keywords:** preoperative, trauma, medications, nil-by-mouth, quality improvement

## Abstract

Introduction

All preoperative trauma patients should receive their preoperative medications regardless of being nil per os (NPO). Anticoagulants, angiotensin-converting enzyme inhibitors (ACE-I), and angiotensin II receptor blockers (ARBs) should be omitted. This is according to both local and national guidelines. We noticed that some preoperative patients have not been receiving their morning medications prior to having their operations. This has led to pre and perioperative complications.

The aim of this study, therefore, was to conduct a quality improvement project to assess if preoperative trauma patients are receiving their prescribed medications preoperatively when placed NPO. We then aimed to determine the adverse outcome associated with omission and to furthermore devise a robust system to prevent recurrence.

Methods

Data were collected from the relevant patients’ drug charts on the day of the operation. These cases were available on the daily trauma list. We evaluated if there was any clear reason documented for not providing the medications. Following the first round, we implemented our action plan for posters^ ^to be taped to drug trolleys on the orthopedic wards and informed the nursing staff of the need to give preoperative medications. We deemed the following to be important medication classes: calcium channel blockers, neuromodulators, beta-blockers, anti-epileptics, digoxin, bronchodilators, anti-anginals, anti-epileptics, and benzodiazepines. This was re-audited after one month to assess compliance and monitor for improvement.

Results

Forty patients were included in the first round and 41 in the re-audit. In the first round, 16/41 (39%) patients received their medications correctly. In patients who did not receive their medications (n=25), 22 had important medication classes omitted. Post implementation of the posters, 25/41 (61%) patients received their medications correctly. In patients who did not receive their medications (n=16), 10 had important medication classes omitted. The main reason why medications were incorrectly not given was that patients were NPO.

Conclusion

This quality improvement audit shows that our interventions between audit cycles have made a significant improvement in patients receiving their medications and therefore this has a direct positive impact on patient safety and outcomes. We should continue to have a close rapport with the nursing staff to maintain standards of correct practice, and these audit findings should be escalated to the emergency theatre thereafter.

## Introduction

Preoperative fasting is important both physiologically and as a precautionary measure during anesthesia and surgery. This involves abstinence from all foods and liquids for a specified period of time before the induction of anesthesia or commencement of surgery [1]. The duration of preoperative fasting is dependent on the type of diet, patient condition, and type of surgery [2].

The main reason for preoperative fasting is to reduce the volume and acidity of stomach contents, therefore decreasing the risk of regurgitation, vomiting, aspiration, and further surgical complications [3]. The Royal College of Nursing guidelines state a minimum fasting period of six hours for food and two hours for clear fluids prior to elective anesthesia or sedation in healthy patients [4].

However, in accordance with national guidelines of the Royal College of Anaesthetists, even though patients must be nil per os (NPO) before theatre, all trauma patients should still receive their prescribed preoperative medications, with only a few exceptions. These include anticoagulants, angiotensin-converting enzyme (ACE) inhibitors, and angiotensin receptor blockers (ARBs) [5].

On conducting a literature review, although there is plenty of research on preoperative fasting, there is a paucity of research pertaining to patients receiving their prescribed medications. The aim of this study, therefore, was to conduct a quality improvement project to assess if preoperative trauma patients are receiving their prescribed medications preoperatively when placed NPO. We then aimed to determine the adverse outcome associated with omission and to furthermore devise a robust system to prevent recurrence.

## Materials and methods

**Figure 1:**
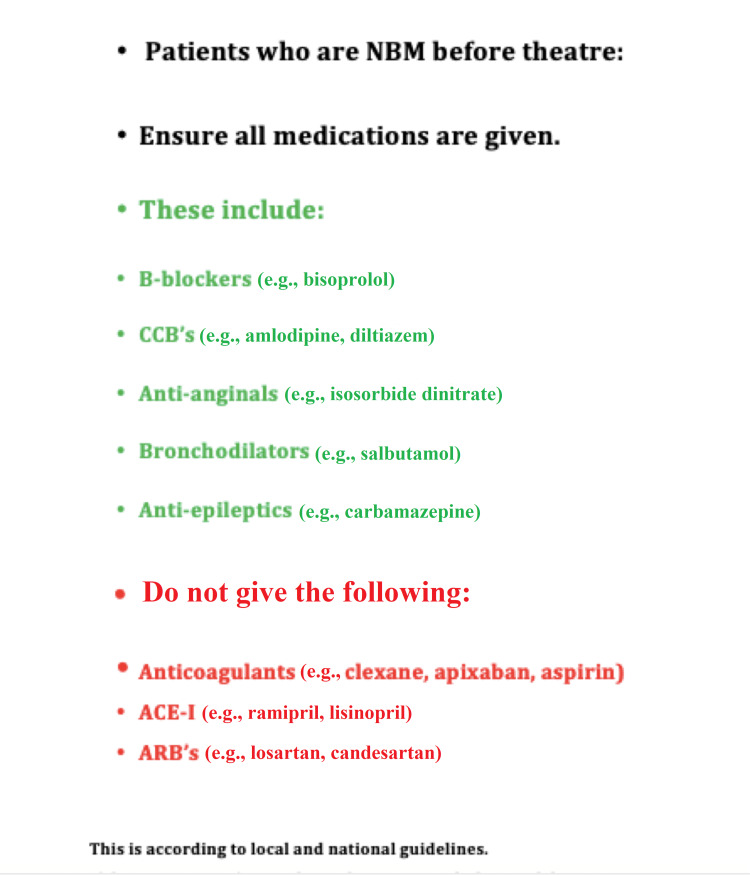
Poster indicating which medications should and should not be given for patients who are NBM before theatre. NBM: nil by mouth; ACE-I: angiotensin-converting enzyme inhibitors; ARB: angiotensin receptor blockers

This single-center prospective study was conducted over a three-week period at Basildon University Hospital to assess if every patient booked onto the trauma theatre list received correct medications preoperatively. Data were extracted by evaluating patients’ notes and drug charts in line with local clinical governance protocols, and IRB approval was obtained. If medications were not given, we assessed if a clear indication was given. Orders for continuing or withholding medications were given by the orthopedic doctors when admitting the patient into hospital, when reviewing the patients on the daily ward round, and during the postoperative period. These orders were documented in writing in the patient notes and also verbally communicated to the ward nurses to minimize errors. Following the initial audit, which included 41 patients, we designed a poster to explain which medications should and should not be given preoperatively. This was taped to the drug trolleys on the surgical wards (Figure 1).

Furthermore, the lead author conducted meetings with the ward managers and the nursing staff to inform them of the correct practice of administering preoperative medications for these NPO patients. We also presented the results at the orthopedic and anesthetic audit meetings. We then conducted a re-audit after one month to assess compliance and monitor improvement. There was a one-year follow-up period. We collected data on the demographic profile, the number of patients who had correctly or incorrectly received their medications, the class of medication in those that were omitted, and the reason for omission.

Inclusion criteria consisted of patients on the trauma theatre list. Exclusion criteria consisted of patients awaiting elective surgery and patients on the emergency theatre list. Patients were required to be NPO for a minimum of six hours. Patients who were last on the list and who had their operation in the late afternoon were allowed to have a light early breakfast depending on their physiological status and co-morbidities. Patients who were first on the list were NPO from midnight.

## Results

The demographic profile of the study populations for the first audit cycle and re-audit cycle is summarized in Table 1 and Table 2, respectively. During the study, a total of 41 patients (33 female, eight male) were included.

**Table 1:** Demographic profile of the study population of the first audit. DHS: dynamic hip screw; IM: intramedullary; ORIF: open reduction internal fixation; ASA: American Society of Anesthesiologists

Total patients (N)		41
Age (years)	Mean	69
Procedure	DHS/IM nail	10 (24.4%)
	Hemi-arthroplasty	9 (22%)
	Ankle ORIF	6 (14.6%)
	Olecranon ORIF	2 (4.9%)
	Washout	6 (14.6%)
	Other	8 (19.5%)
Gender distribution	Male	25 (60.1%)
	Female	16 (39%)
ASA grade	1	16 (39%)
	2	15 (36.6%)
	3	6 (14.6%)
	4	4 (9.8%)

**Table 2:** Demographic profile of the study population for the re-audit cycle. DHS: dynamic hip screw; IM: intramedullary; ORIF: open reduction internal fixation; ASA: American Society of Anesthesiologists; THR: total hip replacement

Total patients (N)		41
Age (years)	Mean	72
Procedure	DHS/IM nail	15 (36.6%)
	Hemi-arthroplasty	11 (26.8%)
	Ankle ORIF	5 (12.2%)
	THR	3 (7.3%)
	Other	7 (17.1%)
Gender distribution	Male	8 (19.5%)
	Female	33 (80.5%)
ASA grade	1	21 (51.2%)
	2	14 (34.1%)
	3	6 (14.6%)

**Figure 2:**
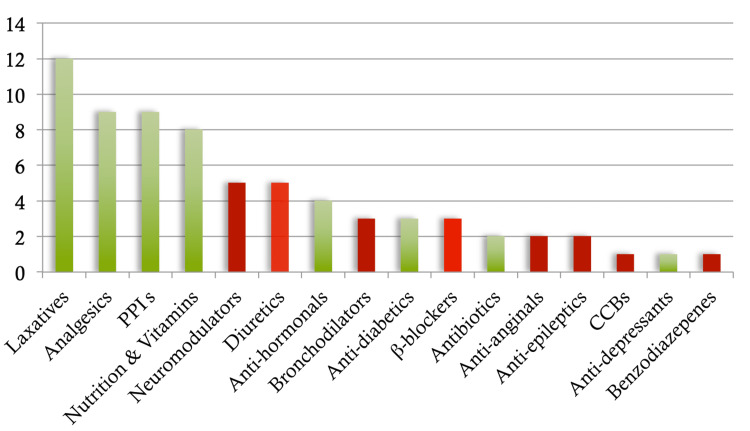
Bar chart showing the medication classes omitted in patients who did not receive their prescribed medications preoperatively from the first audit cycle. PPI: proton pump inhibitors; CCB: calcium channel blockers

From the first audit cycle, only 16 patients (39%) had their preoperative medications given correctly. The medications were not given to 25 patients (61%). The results from the first audit cycle are shown in Figure 2. Figure 2 is a bar chart showing the medication classes omitted in patients who incorrectly did not receive their prescribed medications preoperatively. The medication categories highlighted in red indicate critical drug classes, for which omission may result in dangerous complications for the patient. These were classified as follows: neuromodulators, diuretics, bronchodilators, beta-blockers, calcium channel blockers, anti-angina medications, anti-epileptics, and benzodiazepines. There were 22 critical drug class omissions in the first audit cycle.

**Figure 3:**
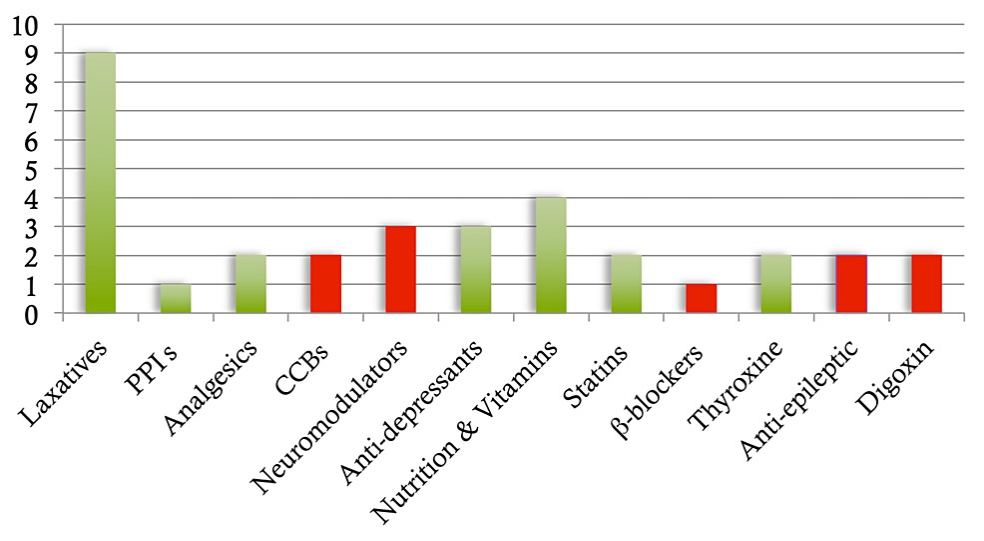
Bar chart showing the medication classes omitted in patients who did not receive their prescribed medications preoperatively from the re-audit cycle. PPI: proton pump inhibitors; CCB: calcium channel blockers

The results from the re-audit cycle are shown in Figure 3. For the re-audit cycle, 25/41 (61%) of patients had their preoperative medications correctly given. This is a significant improvement from the first audit cycle. There were 10 critical drug class omissions. This is a significant improvement compared to the first cycle.

Table 3 demonstrates the reasons shown as to why prescribed medications were not given to patients. From the first audit cycle, 19 patients incorrectly did not receive their prescribed medications because they were NBM. However, after the poster was taped to drug trolleys and meetings conducted with the ward managers, this reduced to 12 patients not being given their medications in the re-audit cycle.

**Table 3:** Reasons for not giving the prescribed medications were compared between the two audit cycles.

Reason	First audit cycle	Re-audit cycle
Nil by mouth	19	12
Refused	3	2
Unavailable	3	2

## Discussion

This study adds value and improves patient safety by assessing whether preoperative trauma patients are indeed receiving their prescribed medications when they are NPO. The key findings of this study are that during the re-audit cycle, there was a significant improvement in patients receiving their preoperative medications compared to the first cycle (61% from 39%). There were also fewer critical drug class medication omissions in the re-audit cycle compared to the first audit (10 compared to 22). We found that the main reason why patients are incorrectly not receiving their prescribed medications was that they were NPO. Our study intervention of applying informative posters onto the drug trolleys and organizing meetings with the nurses and ward managers improved this incorrect perception.

This study assessed whether patients on the trauma list are receiving their prescribed medications when NPO before surgery. Patients who are awaiting surgery on the trauma list for orthopedic trauma operations can be very complex and also suffer from multiple medical co-morbidities. There is a common misconception amongst hospital staff that patients who are NPO should not receive their preoperative medications. The medications which should be omitted include therapeutic anticoagulants, ACE-I, and ARBs [5]. Anticoagulants, in particular DOACs (direct oral anticoagulants) and aspirin, must be omitted due to the increased risk of intraoperative bleeding [6-8]. The American College of Chest Physicians recommends prophylactic low molecular weight heparin to be started 12 or more hours preoperatively due to the increased risk of sustaining a thromboembolic event [9,10]. Angiotensin-converting enzyme inhibitors and ARBs are routinely omitted due to the increased risk of perioperative hypotension, acute kidney injury, and myocardial complications [5]. Our study categorized beta-blockers and CCBs as critical drug-class medications due to the control of heart rate and atrial fibrillation, however, the PeriOperative ISchaemic Evaluation (POISE) trial discourages the use of beta-blocking agents on the day of surgery due to the effects on hypotension and bradycardia [11]. It is therefore important to carefully titrate beta-blockers to a target patient-specific heart rate [12,13].

The findings of our study are also supported by Pearse and Rajakulendran who found that 45% of cardiac medications were not administered before elective surgery [14]. It is clear due to the significant improvement in results from the re-audit cycle that a significant part of the reason why patients are not receiving their medications preoperatively is because of the incorrect assumption that "NPO" means no oral medications. Our intervention of placing posters on the drug trolleys and organizing meetings with the staff on the wards administering the medications had a very positive impact. This demonstrates educating and raising awareness of this issue among the staff members is crucial. In day-to-day clinical practice, staff on the surgical wards sometimes get mixed messages on which medications to withhold and administer for preoperative surgery, but our study aims to standardize and provide clarity on these standards of practice [15,16].

One limitation of this study was that it had a relatively small sample size. However, the sample size of 41 patients per audit cycle (82 patients total) over a three-week period was deemed sufficient for the scope of this study. We also only used patients on the orthopedic trauma list. Future studies can focus on emergency surgery patients and elective patients.

## Conclusions

In conclusion, our study adds value to the literature by highlighting the incorrect assumption that patients who are NPO should not receive their prescribed medications preoperatively. We have shown that our interventions between audit cycles have made a significant improvement in patients receiving their medications and therefore this has a direct positive impact on patient safety and outcomes. We should continue to have a close rapport with the nursing staff to maintain standards of correct practice, and these audit findings should be escalated to the emergency theatre thereafter.
